# Long-term Effectiveness and Predictors of Transdiagnostic Internet-Delivered Cognitive Behavioral Therapy for Emotional Disorders in Specialized Care: Secondary Analysis of a Randomized Controlled Trial

**DOI:** 10.2196/40268

**Published:** 2022-10-31

**Authors:** Alberto González-Robles, Pablo Roca, Amanda Díaz-García, Azucena García-Palacios, Cristina Botella

**Affiliations:** 1 Department of Psychology and Sociology Universidad de Zaragoza Teruel Spain; 2 Department of Psychology Universidad Villanueva Madrid Spain; 3 Department of Basic and Clinical Psychology, and Psychobiology Universitat Jaume I Castellón de la Plana Spain; 4 CIBER Fisiopatología Obesidad y Nutrición (CIBERObn) Instituto Carlos III Madrid Spain

**Keywords:** transdiagnostic, anxiety, depression, long term, predictors

## Abstract

**Background:**

Transdiagnostic internet-delivered cognitive behavioral therapy (iCBT) for emotional disorders has been shown to be effective in specialized care in the short term. However, less is known about its long-term effects in this specific setting. In addition, predictors of long-term effectiveness may help to identify what treatments are more suitable for certain individuals.

**Objective:**

This study aimed to analyze the long-term effectiveness of transdiagnostic iCBT compared with that of treatment as usual (TAU) in specialized care and explore predictors of long-term effectiveness.

**Methods:**

Mixed models were performed to analyze the long-term effectiveness and predictors of transdiagnostic iCBT (n=99) versus TAU (n=101) in public specialized mental health care. Outcomes included symptoms of depression and anxiety, health-related quality of life (QoL), behavioral inhibition and behavioral activation, comorbidity, and diagnostic status (ie, loss of principal diagnosis) from baseline to 1-year follow-up. Sociodemographic characteristics (sex, age, and education) and clinical variables (principal diagnosis, comorbidity, and symptom severity at baseline) were selected as predictors of long-term changes.

**Results:**

Compared with baseline, transdiagnostic iCBT was more effective than TAU in improving symptoms of depression (*b*=–4.16, SE 1.80, 95% CI –7.68 to –0.67), health-related QoL (*b*=7.63, SE 3.41, 95% CI 1.00-14.28), diagnostic status (*b*=–0.24, SE 0.09, 95% CI –1.00 to –0.15), and comorbidity at 1-year follow-up (*b*=–0.58, SE 0.22, 95% CI –1.00 to –0.15). From pretreatment assessment to follow-up, anxiety symptoms improved in both transdiagnostic iCBT and TAU groups, but no significant differences were found between the groups. Regarding the predictors of the long-term effectiveness of transdiagnostic iCBT compared with that of TAU, higher health-related QoL at follow-up was predicted by a baseline diagnosis of anxiety, male sex, and the use of psychiatric medication; fewer comorbid disorders at follow-up were predicted by older age and higher baseline scores on health-related QoL; and fewer depressive symptoms at follow-up were predicted by baseline diagnosis of depression. However, this pattern was not observed for baseline anxiety diagnoses and anxiety symptoms.

**Conclusions:**

The results suggest that transdiagnostic iCBT is more effective than TAU to target depressive symptoms among patients with emotional disorders. Anxiety symptoms remained stable at 1-year follow-up, with no differences between the groups. Results on predictors suggest that some groups of patients may obtain specific gains after transdiagnostic iCBT. Specifically, and consistent with the literature, patients with baseline depression improved their depression scores at follow-up. However, this pattern was not found for baseline anxiety disorders. More studies on the predictor role of sociodemographic and clinical variables in long-term outcomes of transdiagnostic iCBT are warranted. Future studies should focus on studying the implementation of transdiagnostic iCBT in Spanish public specialized mental health care.

**Trial Registration:**

ClinicalTrials.gov NCT02345668; https://clinicaltrials.gov/ct2/show/NCT02345668

## Introduction

### Background

In the past 20 years, epidemiological studies have systematically shown the high prevalence of emotional disorders (ie, anxiety and depression) and the disability and costs associated with these disorders [1-3]. The current COVID-19 pandemic has affected the social, work, and personal functioning of billions of people across the globe [4]. One of the most noteworthy effects of the pandemic has been its impact on mental health, with increases in the prevalence and severity of psychological disorders, including anxiety and depression [5,6]. Because of some social changes wrought by this situation (eg, social distancing), the need for interventions that do not involve face-to-face contact (eg, digital interventions) is growing. Therefore, it is important for people to have access to effective digital interventions such as internet-delivered interventions.

In the field of internet-delivered interventions, most of the research has focused on internet-delivered cognitive behavioral therapy (iCBT). The literature has shown that iCBT for emotional disorders is accessible [7], easy to disseminate [8], safe [9], and effective in both short- and long-term periods. Regarding long-term outcomes, several studies have been carried out that support the long-term effectiveness and efficacy of iCBT at 1-year follow-up [10] or longer periods [11]; for example, Eriksson et al [10] reported 1-year follow-up outcomes of a randomized controlled trial (RCT) that evaluated an iCBT program compared with treatment as usual (TAU) for depression in primary care, with results that supported its effectiveness. In another study, Wootton et al [12] showed that a self-guided iCBT program for obsessive-compulsive disorder (OCD) was effective in reducing OCD symptoms and that these gains were maintained at 1-year follow-up. In sum, research suggests that iCBT also has lasting effects on patients with anxiety and depressive disorders.

Among the range of iCBT programs, transdiagnostic iCBT for emotional disorders has shown its efficacy and effectiveness in a growing number of RCTs. Several meta-analyses have shown that transdiagnostic iCBT is effective in the short term, with pooled effect sizes (Hedges *g*) in the medium-to-large range for overall measures of anxiety (0.78-0.82), depression (0.79-0.84), and quality of life (QoL; 0.48-0.56) after treatment [13,14]. However, most of these available meta-analytic studies mainly report posttreatment outcomes, which suggests that more research is needed on the long-term effects of transdiagnostic iCBT. It is crucial to study not only the short-term effectiveness but also the long-term outcomes of these interventions for several reasons; for instance, high chronicity and relapse rates among anxiety and depressive disorders have commonly been reported in the literature [15,16]. This aspect is directly related to the direct (eg, psychiatric and psychological treatment resources) and indirect (eg, work and social costs) costs associated with the management of these disorders worldwide, which have been reported to be huge [17]. Therefore, it is important to develop interventions that show both short- and long-term effectiveness.

In addition to studying the long-term effectiveness of iCBT, it is essential to study possible predictors because a given treatment is not likely to work in the same way for everyone. Research on predictors of change can help to make recommendations about which treatments are more appropriate for certain individuals and which ones are less likely to be beneficial for them. Specifically, predictors of long-term effectiveness can help to determine for which patients transdiagnostic iCBT is more suitable in the long term. The most frequent predictors in the iCBT arena usually include baseline clinical characteristics (eg, clinical severity at baseline, comorbidity, and diagnosis) [18,19] and sociodemographic variables (eg, age, sex, and education) [20,21]. However, most of the studies conducted in this area have mainly focused on predictors of posttreatment outcomes or short-term follow-up results (eg, 3-month follow-up) [22,23].

### Objectives

To the best of our knowledge, studies on the long-term effectiveness of iCBT in public specialized mental health care are scarce in the literature. However, the high demand for mental health resources as well as the lack of resources in this specific setting [24,25] highlight the need for evidence-based interventions that are also effective in the long term. In a previously published RCT, we examined the effectiveness of a transdiagnostic iCBT compared with that of TAU in public specialized mental health care [26]. Transdiagnostic iCBT was found to be more effective than TAU on measures of anxiety (Cohen *d*=0.35), depression (Cohen *d*=0.41), and health-related QoL (Cohen *d*=–0.45) after treatment. However, the effects of this intervention in the long term (1-year follow-up) have not yet been analyzed. Therefore, the aim of this investigation was 2-fold: to analyze the long-term outcomes of transdiagnostic iCBT for emotional disorders compared with those of TAU and to analyze potential predictors of the long-term effectiveness of transdiagnostic iCBT versus TAU.

## Methods

### Study Design

This study analyzes long-term data (1-year follow-up) from a previously published RCT that compared transdiagnostic iCBT with TAU in public specialized mental health care services [26]. The protocol of the original study was registered at ClinicalTrials.gov (NCT02345668). The study design of the RCT has been fully described elsewhere [27].

### Ethics Approval

This study was granted ethics approval by the ethics committee of Universitat Jaume I (Castellón de la Plana, Spain) and the clinical research ethics committees of the 3 hospitals that participated in the trial (Consorcio Hospitalario Provincial de Castellón, Hospital Universitario de la Ribera, and Hospital Universitario Vall d’Hebron).

### Participants and Procedure

Participants were adults who attended public mental health units in Spain to seek psychological or psychiatric help between July 2015 and June 2019. Potential participants were identified by the psychiatrists and psychologists at these centers and referred to the study researchers for eligibility assessment (refer to the study by González-Robles et al [26] for a full description of the recruitment process). To participate in the study, patients had to meet the following eligibility criteria: (1) aged ≥18 years; (2) ability to understand and read Spanish; (3) access to the internet at home and an email address; (4) fulfill Diagnostic and Statistical Manual of Mental Disorders, Fourth Edition, Text Revision, diagnostic criteria [28] for emotional disorders, including major depressive disorder, dysthymic disorder, depression not otherwise specified, panic disorder, agoraphobia, social anxiety disorder, generalized anxiety disorder, anxiety not otherwise specified, and OCD; (5) provide written informed consent; (6) absence of schizophrenia, bipolar disorder, and alcohol or substance dependence disorder; (7) absence of high risk of suicide; (8) absence of a disabling medical disease that prevented the participant from undergoing psychological treatment; and (9) not receiving another psychological treatment during the study (in the experimental group). Pharmacological treatment was allowed, but participants had to be taking the same dose during the 2 months before enrolling in the study. In addition, participants in the experimental group whose medication was increased or changed during the study period were excluded from the trial (decreases in pharmacological treatment were accepted).

**Figure 1:**
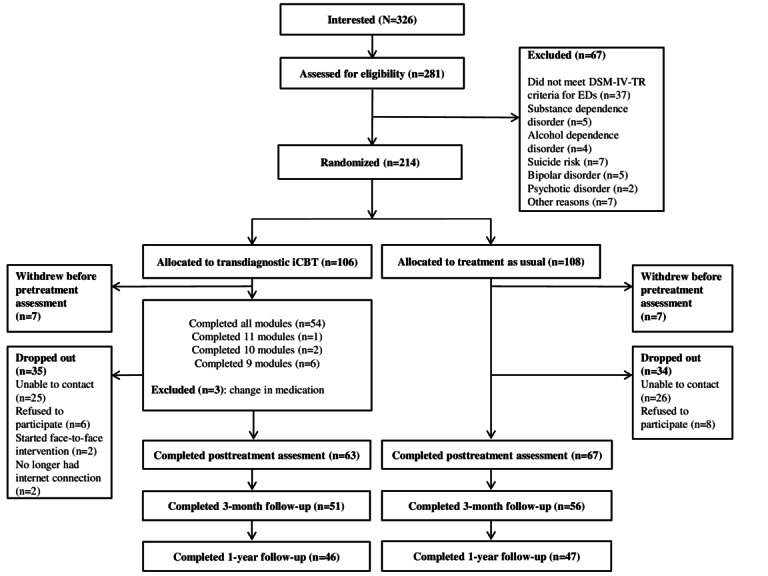
Flowchart of participants. DSM-IV-TR: Diagnostic and Statistical Manual of Mental Disorders, Fourth Edition, Text Revision; ED: emotional disorder; iCBT: internet-delivered cognitive behavioral therapy.

The flowchart of participants from baseline to 1-year follow-up is presented in Figure 1. A total of 326 patients expressed interest in the study, of whom 281 (86.2%) were assessed for eligibility. Of these 281 participants, 67 (23.8%) were excluded from the study, and the remaining 214 (76.2%) participants were randomized to either transdiagnostic iCBT (n=106, 49.5%) or TAU (n=108, 50.5%). In addition, a few (transdiagnostic iCBT: 7/106, 6.6%; TAU: 7/108, 6.5%) patients withdrew from the study before the pretreatment assessment; therefore, they were excluded from the analyses. Thus, of the 200 participants in the final sample at baseline, 99 (49.5%) were randomized to transdiagnostic iCBT and 101 (50.5%) to TAU. One-year follow-up data were obtained from 47% (46/99) of the participants in the transdiagnostic iCBT condition and 47% (47/101) of the participants in the TAU condition.

### Instruments

#### Principal Outcomes

The Beck Depression Inventory, Second Edition (BDI-II) [29] is a self-report scale consisting of 21 items about the symptoms that characterize major depressive disorder. Scores on each item range from 0 to 3, and the maximum score is 63 points. The instrument has demonstrated internal consistency in both the original version (Cronbach *α*=.76-.95) and the Spanish version (Cronbach *α*=.87-.89) [30]. The Cronbach *α* value for the BDI-II in this study at baseline was .90.

The Beck Anxiety Inventory (BAI) [31] is a 21-item self-report questionnaire that assesses anxiety, with a maximum score of 63 points. Each item has a 4-point severity scale (from 0=not at all to 3=severely) that addresses anxiety symptoms experienced during the previous week. Several validation studies have shown adequate psychometric properties, with good-to-excellent internal consistency (Cronbach *α* between .85 and .94) and convergent and divergent validity. The Spanish version of the BAI has demonstrated high internal consistency (Cronbach *α*=.93) [32]. The Cronbach *α* value for the BAI in this study at baseline was .92.

#### Secondary Outcomes

The EQ-5D-3L [33] is a generic instrument that measures health-related QoL and consists of 2 parts. Part 1 assesses self-reported problems in each of the following 5 domains: mobility, self-care, daily activities, pain or discomfort, and anxiety or depression. Part 2 records the participant’s self-assessed health on a visual analog scale, a 10-cm vertical line on which the best and worst imaginable health states score 100 and 0, respectively. In this study, health-related QoL was assessed using the visual analog scale.

The behavioral inhibition system (BIS) and behavioral activation system (BAS) scales [34,35] contain 20 items rated from 1 to 4, with 7 BIS subscale items that evaluate individuals’ emotional responses to impending negative events and 13 BAS subscale items that evaluate individuals’ behavioral and emotional responses to potentially positive events. The BIS and BAS scales have shown good reliability in individuals with emotional disorders (Cronbach *α*=.73-.92) and good convergent and divergent validity as indicators of temperament. The internal consistency of the Spanish version ranges between Cronbach *α*=.65 and Cronbach *α*=.82. The Cronbach *α* values for the BIS and BAS subscales in this study at baseline were .61 and .80, respectively.

### Interventions

#### Transdiagnostic iCBT

All participants received a 12-module transdiagnostic iCBT protocol through the web platform designed by our research group [36]. The core components of the treatment are based on the unified protocol [37,38] and some treatment strategies from dialectical behavioral therapy (eg, what and how techniques) [39]. Participants are trained to learn and practice adaptive transdiagnostic iCBT skills through the following components: (1) present-focused emotional awareness (modules 4 and 5), (2) cognitive flexibility (modules 6 and 7), (3) identification and modification of emotional avoidance patterns and emotion-driven behaviors (modules 8 and 9), and (4) exposure (interoceptive and situational; modules 10 and 11). The treatment contains 4 additional modules: an introduction module (module 1), a module to facilitate the patient’s engagement with the therapy (module 2), a module with psychoeducation on emotions (module 3), and a relapse prevention module at the end of the treatment (module 12). In addition, transdiagnostic iCBT includes a module 0 (welcome module) with information and recommendations about how to use the protocol. The modules are presented sequentially to enable step-by-step movement through the program. All participants had access to the protocol for a maximum period of 18 weeks, and they were allowed to use the program any time they wanted to during the trial period (ie, including the follow-up periods). Additional details about this treatment have been reported elsewhere [26,27]. The treatment modules and their goals are depicted in Textbox 1.

**Treatment modules and their goals.**

- 0. Welcome module: gives information about the protocol (eg, general aim and number of modules) and recommendations about how to use the treatment protocol
- 1. Introduction to treatment: provides a framework on the role of emotion regulation in emotional disorders
- 2. Motivation for change: promotes patients’ motivation for change by analyzing the pros and cons of changing and trains them in goal setting
- 3. Understanding the role of emotions: provides psychoeducation on the nature, role, and functions of emotions and trains the patient in identifying emotion components
- 4. Nonjudgmental emotional awareness and acceptance of emotional experiences: trains the patient in nonjudgmental emotional awareness and the acceptance of emotional experiences
- 5. Practicing present-focused awareness: provides additional training in nonjudgmental present-focused awareness in different domains, namely physical sensations, thoughts, emotions, and daily activities
- 6. Learning to be flexible: teaches the patient how to identify maladaptive cognitive patterns (ie, thinking traps)
- 7. Practicing cognitive flexibility: trains the patient in modifying maladaptive cognitive patterns (ie, cognitive reappraisal) and also provides psychoeducation to the patient about intrusive thoughts and how to manage them
- 8. Emotional avoidance: trains the patient to understand and identify maladaptive emotion avoidance patterns
- 9. Emotion-driven behaviors (EDBs): patients learn the concept of EDB and to replace maladaptive EDB with other more adaptive behaviors (ie, opposite action)
- 10. Accepting and facing physical sensations: teaches patients about the role of physical sensations in emotional response and trains them in interoceptive exposure
- 11. Facing emotions in the contexts where they occur: trains the patient to face situation-elicited avoided emotions through exposure procedures
- 12. Relapse prevention: patients review what they have learned throughout the program, schedule future practice of skills, and learn how to cope with high-risk situations

#### TAU Provision

TAU was delivered by psychiatrists and clinical psychologists at mental health units in Spain. TAU in this study was provided by 3 hospitals: Consorcio Hospitalario Provincial de Castellón (Castellón de la Plana), Hospital Universitario de la Ribera (Valencia), and Hospital Universitario Vall d’Hebron (Barcelona). To maximize the external validity of this RCT, participants in this condition were allowed to receive psychiatric treatment (ie, prescription and monitoring of antidepressant and anxiolytic medication), psychological treatment (including case management, group psychotherapy, empathic listening, and supportive counseling), or a combination of the two. The frequency of visits during the 18-week treatment period varied depending on the type of treatment (ie, psychiatric or psychological) provided to the participant. Patients in the TAU condition who were already receiving any of the aforementioned treatments at the time of enrollment were informed that they would continue to receive these services during the treatment period. Furthermore, participants who were receiving any treatment other than those provided at the mental health unit were excluded from the trial.

### Statistical Analyses

First, independent samples 2-tailed *t* tests for continuous data and chi-square tests for categorical data were performed to confirm that there were no demographic or clinical differences between transdiagnostic iCBT and TAU at baseline. Missing data were explored, and we concluded that 33.63% of the values were missing overall at the construct level. The Little missing completely at random test showed that data were missing completely at random (*χ*^2^_80_=76.1; *P*=.60). Furthermore, completers did not differ significantly from dropout cases on the baseline variables: age (*t*_198_=1.23; *P*=.22), sex (*c*^2^_1_=0.02; *P*=.90), marital status (*c*^2^_3_=0.28; *P*=.96), and education (*c*^2^_2_=2.21; *P*=.33). Multiple imputation of missing values is not necessary before performing longitudinal mixed model analysis [40].

Mixed effects models were conducted to analyze the long-term effects and predictors of transdiagnostic iCBT using the *lmer* function from the *lme4* R package [41] with R software (version 4.0.2; The R Foundation for Statistical Computing) [42]. Analyses were conducted via restricted maximum likelihood estimation [43,44]. In contrast to multiple imputation methods (ie, to fill in missing data) or complete case data through detection methods (which result in biased estimations), the restricted maximum likelihood estimation method allows incomplete and unbalanced data to be modeled by finding parameters that maximize the likelihood using all the available data, providing a less biased estimate of variance components with smaller sample sizes [43,45,46]. To compute the magnitude of between-group changes at 1-year follow-up, effect sizes (Cohen *d*) were calculated by dividing the differences in means by the pooled SD. Effect sizes were interpreted according to the Cohen convention: effect sizes of 0.20 are considered low, effect sizes of 0.50 are considered medium, and effect sizes of ≥0.80 are considered large [47].

Mixed effects models were performed on 5 dependent variables (DVs): depression (ie, BDI-II score), anxiety (ie, BAI score), BIS (ie, BIS scale score), BAS (ie, BAS scale score), health-related QoL (ie, EQ-5D-3L score), comorbidity (ie, number of diagnoses), and diagnostic status (ie, dummy variable: 0=does not meet diagnostic criteria and 1=meets diagnostic criteria). The models had 2 main components: fixed effects and random effects. First, variance across participants and hospitals was modeled as random effects in the model (ie, participant and hospital as random effects that account for individual differences in DVs). Second, group (ie, transdiagnostic iCBT vs TAU) and time (ie, pretreatment assessment, posttreatment assessment, 3-month follow-up, and 1-year follow-up) were modeled as fixed effects. Furthermore, we selected different baseline variables as potential predictors of long-term outcomes, including demographic variables (ie, sex, 0=male and 1=female; age, and education, 0=nonuniversity [basic and medium studies] and 1=university), clinical status (ie, medication. 0=no and 1=yes; principal diagnosis, 0=depression, 1=anxiety, and 2=OCD; comorbidity, number of clinical diagnoses, and diagnostic status, 0=does not meet diagnostic criteria and 1=meets diagnostic criteria), dispositional traits (ie, behavioral inhibition and behavioral activation), and symptomatology (anxiety [BAI], depression [BDI-II], and health-related QoL [EQ-5D-3L]). Given that we were interested in long-term changes (ie, 1-year follow-up compared with baseline), we focused on group1×time4 interactions (ie, transdiagnostic iCBT×1-year follow-up) to analyze the long-term effects and predictors [48].

Specification of the random effect structure was modeled following the recommendations by Barr et al [49,50]. First, a null model was estimated as a baseline point of comparison (ie, model 0). The null models only included the random intercepts (ie, participant and hospital) without the fixed effects. Second, models were computed by testing the long-term effects on the different DVs (ie, model 1). Third, a 2-step approach was used to analyze the long-term predictors [51,52]: first, univariate mixed models were computed to investigate the independent contribution of each potential predictor separately (ie, model 2); next, significant predictors in univariate models (ie, group1×time4×predictor; *P*<.05) were entered simultaneously into the multivariate mixed models using backward deletion (ie, model 3). This procedure helped us to obtain a final adjusted model for each DV.

Effect sizes for each model are presented as the model-derived fixed-effect parameter regression weights [53,54]. Model fit was evaluated using the likelihood ratio test, Akaike information criteria, and Bayesian information criteria.

## Results

### Baseline Data

The participants (N=200) had a mean age of 38.64 (SD 10.61; range 18-68) years, and the majority were female (138/200, 69%). Table 1 shows the sociodemographic and clinical characteristics for both conditions at baseline. There were no significant differences between transdiagnostic iCBT and TAU at baseline on any of the sociodemographic and clinical characteristics. Moreover, no significant differences were found for medication, principal diagnosis, number of comorbid diagnoses, or clinical severity on any of the measures.

**Table 1:** Sociodemographic and clinical characteristics of the sample at baseline (N=200).

Characteristic		Transdiagnostic iCBT^a^ (n=99)	TAU^b^ (n=101)	Chi-square (*df*)		*t* test (*df*)		*P* value	
Age (years), mean (SD)		38.64 (10.61)	38.25 (11.03)	N/A^c^		0.25 (1)		.80	
**Sex, n (%)**					1.3 (1)		N/A		.26
	Female	72 (72.7)	66 (65.3)						
	Male	27 (27.3)	35 (34.7)						
**Marital status, n (%)**					1.1 (3)		N/A		.78
	Single	22 (22.2)	26 (25.7)						
	Married or partnered	63 (63.6)	65 (64.4)						
	Divorced or widowed	14 (14.1)	10 (9.9)						
**Education,** **n (%)**					2.1 (2)		N/A		.35
	Basic studies	26 (26.3)	36 (35.6)						
	Secondary studies	41 (41.4)	35 (34.6)						
	University studies	32 (32.3)	30 (29.7)						
**Principal diagnosis, n (%)**					2.7 (8)		N/A		.95
	GAD^d^	23 (23.2)	26 (25.7)						
	AG^e^	16 (16.2)	13 (12.9)						
	PD^f^	9 (9.1)	5 (5)						
	SAD^g^	4 (4)	4 (4)						
	OCD^h^	8 (8.1)	12 (11.9)						
	MDD^i^	20 (20.2)	22 (21.8)						
	DD^j^	7 (7.1)	6 (5.9)						
	Anxiety NOS^k^	10 (10.1)	9 (8.9)						
	Depression NOS	2 (2)	3 (3)						
**Comorbidity,** **n (%)**					2.3 (3)		N/A		.50
	1	49 (49.5)	41 (40.6)						
	2	29 (29.3)	38 (37.6)						
	3	15 (15.2)	13 (12.9)						
	≥4	6 (6.1)	8 (7.9)						
**Medication,** **n (%)**					5.2 (3)		N/A		.16
	None	29 (29.3)	18 (17.8)						
	Antidepressant	22 (22.2)	20 (19.8)						
	Anxiolytic	10 (10.1)	17 (16.8)						
	Both	38 (38.4)	46 (45.5)						

^a^iCBT: internet-delivered cognitive behavioral therapy.

^b^TAU: treatment as usual.

^c^N/A: not applicable.

^d^GAD: generalized anxiety disorder.

^e^AG: agoraphobia.

^f^PD: panic disorder.

^g^SAD: social anxiety disorder.

^h^OCD: obsessive-compulsive disorder.

^i^MDD: major depressive disorder.

^j^DD: dysthymic disorder.

^k^NOS: not otherwise specified.

### Long-term Effectiveness

#### Principal and Secondary Outcomes

Table 2 displays the means, SEs, and effect sizes for transdiagnostic iCBT versus TAU for depression and anxiety symptoms, health-related QoL, and behavioral inhibition and behavioral activation from baseline to 1-year follow-up.

Regarding depressive symptoms, there was a significant main effect of time (*b*=–5.85, SE 1.26, 95% CI –8.30 to –3.40), indicating that BDI-II scores were, on average, 5.85 points greater at baseline than at 1-year follow-up when the other variables in the model were kept constant. The main effect of group was not significant (*b*=–0.71, SE 1.76, 95% CI –4.04 to 2.85); thus, both groups showed similar BDI-II scores on average. The results also revealed a significant group×time interaction (*b*=–4.16, SE 1.80, 95% CI –7.68 to –0.67). Tukey-corrected post hoc comparisons indicated that both groups significantly reduced their depressive symptoms at 1-year follow-up; however, these reductions were larger in the transdiagnostic iCBT group.

Regarding anxiety symptoms, there was a significant main effect of time (*b*=–5.37, SE 1.42, 95% CI –8.13 to –2.61), indicating that BAI scores were, on average, 5.37 points greater at baseline than at 1-year follow-up when the other variables in the model were kept constant. The main effect of group was not significant (*b*=2.67, SE 1.73, 95% CI –5.64 to 1.11); thus, both groups showed similar BAI scores on average. The group×time interaction was not significant (*b*=–1.54, SE 2.03, 95% CI –5.49 to 2.41). Tukey-corrected post hoc comparisons indicated that both groups similarly reduced their anxiety symptoms at 1-year follow-up.

Regarding health-related QoL, there was a significant main effect of time (*b*=6.19, SE 2.38, 95% CI 1.54-10.83), indicating that EQ-5D-3L scores were, on average, 6.19 points greater at 1-year follow-up than at baseline when the other variables in the model were kept constant. The main effect of group was not significant (*b*=2.29, SE 2.59, 95% CI –2.75 to 7.34); thus, both groups showed similar EQ-5D-3L scores on average. The results also revealed a significant group×time interaction (*b*=7.63, SE 3.41, 95% CI 1.00-14.28). Tukey-corrected post hoc comparisons indicated that only transdiagnostic iCBT participants significantly improved health-related QoL at 1-year follow-up, whereas no changes were found in the TAU group.

Regarding the BIS scale, the time main effect (*b*=0.24, SE 0.45, 95% CI –.64 to 1.13) and group main effect (*b*=–0.07, SE 0.43, 95% CI –0.92 to 0.77) were not significant. The results revealed a significant group×time interaction (*b*=–2.37, SE 0.65, 95% CI –3.63 to –1.10). Tukey-corrected post hoc comparisons indicated that only participants in the transdiagnostic iCBT group significantly reduced BIS scale scores at 1-year follow-up, whereas no changes were found in the TAU group. Regarding the BAS scale, the time main effect (*b*=–1.08, SE 0.67, 95% CI –2.38 to 0.23), group main effect (*b*=–0.58, SE 0.87, 95% CI –2.28 to 1.12), and group×time interaction (*b*=1.84, SE 0.96, 95% CI –0.03 to 3.71) were not significant.

**Table 2:** Means, SEs, and between-group effect sizes (Cohen *d*) from baseline to 1-year follow-up.

	Baseline		One-year follow-up		
	Transdiagnostic iCBT^a^, mean (SE)	TAU^b^, mean (SE)	Transdiagnostic iCBT, mean (SE)	TAU, mean (SE)	Cohen *d* (95% CI)
BDI-II^c^	23.4 (1.4)	24.1 (1.4)	13.4 (1.7)	18.3 (1.6)	0.43 (0.02 to 0.85)
BAI^d^	20.0 (1.3)	22.3 (1.2)	13.1 (1.6)	16.9 (1.6)	0.34 (–0.07 to 0.75)
EQ-5D-3L	55.8 (2.0)	53.5 (1.9)	69.6 (2.6)	59.7 (2.5)	–0.57 (–0.98 to –0.15)
BIS^e^ scale	23.3 (0.3)	23.4 (0.3)	21.2 (0.4)	23.6 (0.4)	0.87 (–1.30 to –0.45)
BAS^f^ scale	35.3 (0.6)	35.8 (0.6)	36.0 (0.8)	34.8 (0.8)	0.22 (–0.19 to 0.63)

^a^iCBT: internet-delivered cognitive behavioral therapy.

^b^TAU: treatment as usual.

^c^BDI-II: Beck Depression Inventory, Second Edition.

^d^BAI: Beck Anxiety Inventory.

^e^BIS: behavioral inhibition system.

^f^BAS: behavioral activation system.

#### Diagnostic Status and Comorbidity

Regarding diagnostic status (ie, loss of baseline principal diagnosis), there was a significant main effect of time (*b*=–0.54, SE 0.07, 95% CI –0.86 to –0.20), indicating that changes in diagnostic status were significant at 1-year follow-up compared with baseline when the other variables in the model were kept constant. However, the main effect of group was not significant (*b*=0.00, SE 0.05, 95% CI –0.37 to 0.18); thus, both groups showed similar changes in diagnostic status. The results also revealed a significant group×time interaction (*b*=–0.24, SE 0.09, 95% CI –1.00 to –0.15). Tukey-corrected post hoc comparisons indicated that patients in both groups significantly improved their diagnostic status at 1-year follow-up; however, these changes were significantly higher in the transdiagnostic iCBT group. Specifically, 22% (22/99) of the patients in the transdiagnostic iCBT group met the diagnostic criteria at 1-year follow-up versus 45.5% (46/101) of the patients in the TAU group.

Finally, regarding comorbidity (ie, number of Diagnostic and Statistical Manual of Mental Disorders, Fourth Edition, Text Revision, diagnoses), there was a significant main effect of time (*b*=–0.53, SE 0.17, 95% CI –0.86 to –0.20), indicating that comorbidity was, on average, 0.53 points greater at baseline than at 1-year follow-up when the remaining variables in the model were kept constant. However, the main effect of group was not significant (*b*=–0.10, SE 0.14, 95% CI –0.37 to 0.18); thus, both groups showed similar comorbidity on average. The results also revealed a significant group×time interaction (*b*=–0.58, SE 0.22, 95% CI –1.00 to –0.15). Tukey-corrected post hoc comparisons indicated that both groups significantly reduced comorbidity at 1-year follow-up; however, these reductions were larger in the transdiagnostic iCBT group. Specifically, comorbidity decreased from 1.78 (SE 0.1) diagnoses at baseline in the transdiagnostic iCBT group to 0.70 (SE 0.1) at 1-year follow-up. By contrast, comorbidity in the TAU group changed from 1.88 (SE 0.1) diagnoses at baseline to 1.38 (SE 0.2) at 1-year follow-up.

### Predictors of Long-term Effectiveness

#### Overview

Fixed-effect parameter estimates and their corresponding 95% CIs for each predictor of long-term changes separately are shown in Multimedia Appendix 1. As indicated previously, we first conducted univariate mixed models to investigate the independent contribution of each potential predictor of long-term changes separately. Significant predictors in univariate models were then entered simultaneously into a multivariate mixed model. In the following paragraphs, we report the results on predictors for the following variables: (1) depressive and anxiety symptoms as well as behavioral inhibition and behavioral activation, (2) health-related QoL, (3) diagnostic status, and (4) comorbidity.

#### Changes in Depression, Anxiety, and Behavioral Inhibition and Behavioral Activation

The principal diagnosis (anxiety: *b*=9.07, SE 3.99, 95% CI 1.15-16.72; OCD: *b*=15.97, SE 7.25, 95% CI 2.10-29.88) and comorbidity (*b*=5.14, SE 2.13, 95% CI 1.05-9.22) at baseline predicted changes in depressive symptoms at 1-year follow-up. However, none of the predictors were significantly related to changes in anxiety symptoms, behavioral inhibition, or behavioral activation. Post hoc comparisons of the principal diagnosis at baseline showed the following results: (1) there were significant differences in depressive symptoms at follow-up between transdiagnostic iCBT and TAU only in participants with depressive disorders at baseline and not in participants with anxiety and OCD diagnoses. Specifically, participants with depressive disorders showed significant decreases in depressive symptoms at follow-up in the transdiagnostic iCBT group, whereas no changes were found in the TAU group; (2) participants with anxiety disorders showed significant reductions in depressive symptoms at 1-year follow-up in both transdiagnostic iCBT and TAU groups; (3) however, no changes in depressive symptoms were found in participants with OCD; and (4) participants with depressive disorders in the transdiagnostic iCBT group showed greater depressive symptoms at baseline than participants with anxiety and OCD diagnoses, but these differences disappeared at follow-up. However, these differences were not found in the TAU group. By contrast, transdiagnostic iCBT participants showed a significant positive association between comorbidity and depressive symptoms both at baseline and at 1-year follow-up (ie, participants with severe depressive symptoms showed greater comorbidity), but this association was significantly stronger in the transdiagnostic iCBT group at follow-up, and it was not significant in the TAU group. Finally, when these predictors were entered simultaneously into the mixed model, a principal diagnosis of anxiety at baseline (*b*=11.17, SE 5.66, 95% CI 0.61-21.88) and comorbidity (*b*=5.40, SE 2.13, 95% CI 1.37-9.38) remained significant, whereas a principal diagnosis of OCD at baseline was no longer significant (*b*=14.35, SE 10.01, 95% CI –4.39 to 33.26). The final model significantly improved the model fit (*χ*^2^_7_=111.6; *P*<.001).

#### Changes in Health-Related QoL

Sex (*b*=16.40, SE 7.19, 95% CI 2.48-30.28), medication (*b*=17.99, SE 8.82, 95% CI 0.95-35.04), and a principal diagnosis of anxiety at baseline (*b*=–15.76, SE 7.61, 95% CI –30.33 to –1.10) predicted changes in health-related QoL at 1-year follow-up. Post hoc comparisons showed that there were significant differences in health-related QoL at follow-up between the transdiagnostic iCBT group and the TAU group in male participants but not in female participants. Specifically, significant health-related QoL improvements at follow-up were found in the male participants in the transdiagnostic iCBT group but not in the male participants in the TAU group. Similarly, significant differences in health-related QoL between transdiagnostic iCBT and TAU were found only in participants who were taking medications at baseline. Specifically, significant health-related QoL improvements at follow-up were found in participants taking medications in the transdiagnostic iCBT group but not in the TAU group. Finally, post hoc comparisons showed that only participants with an anxiety diagnosis in the transdiagnostic iCBT group experienced significant increases in health-related QoL at follow-up, whereas no changes were found in the TAU group. Moreover, when these predictors were entered simultaneously into the mixed model, sex (*b*=14.45, SE 7.20, 95% CI 0.88-27.98) and a principal diagnosis of anxiety at baseline (*b*=–14.86, SE 7.67, 95% CI –29.37 to –0.46) remained significant, whereas medication was no longer significant (*b*=15.09, SE 8.93, 95% CI –1.69 to 31.96). The final model significantly improved the model fit (*χ*^2^_32_=64.3; *P*<.001).

#### Changes in Diagnostic Status

Age (*b*=–0.02, SE 0.01, 95% CI 0.03-0.001) and health-related QoL (*b*=–0.01, SE 0.01, 95% CI –0.02 to –0.001) predicted changes in diagnostic status at follow-up. First, there was a negative association between age and diagnostic status in the transdiagnostic iCBT group at follow-up (ie, older people had fewer diagnoses at follow-up), whereas this relationship was positive in the TAU group (ie, older people had more diagnoses at follow-up). Second, there was a significant negative association between health-related QoL and diagnostic status at follow-up in the transdiagnostic iCBT group (ie, participants with better health-related QoL had fewer diagnoses at follow-up). However, this association was not significant in the TAU group. Finally, when these predictors were entered simultaneously into the mixed model, health-related QoL remained significant (*b*=–0.01, SE –0.01, 95% CI –0.02 to –0.001), whereas age was no longer significant (*b*=–0.01, SE –0.01, 95% CI –0.02 to –0.01). The final model significantly improved the model fit (*χ*^2^_7_=296.4; *P*<.001).

#### Changes in Comorbidity

Age (*b*=–0.04, SE 0.02, 95% CI –0.08 to 0.000), education (*b*=0.89, SE 0.49, 95% CI –0.004 to 1.83), and health-related QoL (*b*=–0.02, SE 0.01, 95% CI –0.04 to 0.000) were marginally significant predictors of comorbidity changes at 1-year follow-up. First, age and comorbidity at follow-up were negatively associated in the transdiagnostic iCBT group but not in the TAU group (ie, older people had less comorbidity). Second, no significant differences in comorbidity were found between participants with nonuniversity and university education in the transdiagnostic iCBT group; that is, both participants with nonuniversity and university education significantly reduced their comorbidity in this group. Moreover, participants with nonuniversity education in the transdiagnostic iCBT group had less comorbidity than participants with nonuniversity education in the TAU group at follow-up. Third, a significant negative association between comorbidity and health-related QoL was found in the transdiagnostic iCBT group at follow-up (ie, higher scores in health-related QoL were associated with less comorbidity), but this association was not significant in the TAU group. Finally, when these predictors were entered simultaneously into the mixed model, only health-related QoL remained significant (*b*=–0.03, SE 0.02, 95% CI –0.05 to –0.04), whereas age (*b*=–0.03, SE 0.02, 95% CI –0.08 to 0.01) and education (*b*=0.91, SE 0.51, 95% CI –0.05 to 1.89) were no longer significant. The final model significantly improved the model fit (*χ*^2^_7_=127.5; *P*<.001).

## Discussion

### Overview

The objective of this study was 2-fold. The first objective was to analyze the long-term effectiveness of a transdiagnostic iCBT protocol for emotional disorders in public specialized mental health care services. In addition, we were interested in examining potential predictors of long-term outcomes. We discuss the findings in the next 2 sections.

### Long-term Effectiveness

Taken together, transdiagnostic iCBT was shown to be more effective than TAU at 1-year follow-up for the treatment of emotional disorders in specialized care. Specifically, in comparison with TAU, transdiagnostic iCBT was more effective in reducing symptoms of depression and improving health-related QoL at 1-year follow-up, with effect sizes in the small-to-moderate range. Furthermore, participants in the transdiagnostic iCBT group had a better diagnostic status (ie, of the 99 patients in the transdiagnostic iCBT group, 22, 22%, met the diagnosis criteria at 1-year follow-up, whereas of the 101 patients in the TAU group, 46, 45.5%, met the diagnosis criteria at 1-year follow-up) and showed less comorbidity (0.7 diagnoses in the transdiagnostic iCBT group vs 1.38 diagnoses in the TAU group). Regarding anxiety, both groups improved their scores from pretreatment assessment to 1-year follow-up, without significant differences between the groups. These results suggest that transdiagnostic iCBT was at least as effective as TAU for anxiety symptoms in the long term. In this regard, it should be noted that participants in the TAU group were also undergoing some treatment (pharmacological or psychological treatment) provided by clinicians of the national health system, that is, patients in the TAU group received an active treatment. As a recent meta-analysis on the efficacy of the unified protocol shows, studies that used active control conditions (including TAU) as comparison groups exhibited lower effect sizes than those that used passive control groups (eg, waitlist control group) [55]. Moreover, we do not know how many of the patients in the TAU group continued to receive treatment between follow-up periods (and the types of treatments they received), which might have also influenced the follow-up results. In any case, although the results go in this direction, this hypothesis should be tested using an appropriate study design (eg, noninferiority trial design). Another noteworthy finding is that the scores on behavioral inhibition were significantly lower in patients from the transdiagnostic iCBT condition, with an effect size in the large range (Cohen *d*=0.87), whereas no differences were found for behavioral activation. The study of how these and other related dimensions (eg, neuroticism and extraversion as well as positive and negative affect) change after treatment is of paramount importance in the context of transdiagnostic treatments that target shared psychopathological processes [55,56]. Nevertheless, these results are comparable to those obtained in trials that measure close constructs such as positive and negative affect [57]. The pattern of results that we obtained in behavioral inhibition and behavioral activation resembles the pattern of results in negative and positive effects after transdiagnostic iCBT found in the literature, that is, large gains in negative affect and low-to-moderate gains in positive affect [55]. With the exception of the study by Carl et al [58], to our knowledge, no other studies on transdiagnostic iCBT have included this measure in their trials. Future research may benefit from including measures of behavioral inhibition and behavioral activation to study the potential of transdiagnostic iCBT in successfully addressing these specific temperament dimensions.

Overall, the results support the long-term effectiveness of transdiagnostic iCBT in comparison with that of TAU for a number of measures, including depression, health-related QoL, behavioral inhibition, diagnostic status, and comorbidity. These results add to those of our previously published RCT, which showed that transdiagnostic iCBT in public specialized care was both effective and safe in the short term for individuals with emotional disorders [26]. Improvements in anxiety symptoms remained stable at 1-year follow-up, with no difference between the conditions. However, it should be noted that transdiagnostic iCBT was compared with an active treatment condition, where most patients were receiving either pharmacological or psychological treatment.

To our knowledge, this is the first study to report on the long-term effectiveness of transdiagnostic iCBT for emotional disorders in public specialized mental health care and support its application in this setting. On the basis of our results, we believe that the implementation of iCBT in public specialized mental health care might help to deal with long-lasting barriers (eg, lack of resources) and the difficulties arising from the current COVID-19 pandemic by increasing dissemination of, and access to, evidence-based interventions.

### Predictors of Long-term Effectiveness

#### Overview

The second goal of the study was to analyze potential predictors of long-term effectiveness. To do so, we explored the predictor role of different baseline variables, including sociodemographic characteristics (ie, age, sex, and education) and clinical variables (ie, symptom severity, principal diagnosis, and comorbidity) in the following outcomes at 1-year follow-up: depressive symptoms, anxiety symptoms, health-related QoL, behavioral inhibition and behavioral activation, comorbidity (ie, number of diagnoses), and diagnostic status (ie, loss of principal diagnosis). Overall, a heterogeneous pattern of predictors of long-term outcomes was observed, but we found several relationships that are worth discussing.

#### Changes in Depression, Anxiety, and Behavioral Inhibition and Behavioral Activation

We found that participants in the transdiagnostic iCBT group with a baseline diagnosis of depression had fewer depressive symptoms at follow-up. In addition, participants with depressive disorders at baseline in the transdiagnostic iCBT group showed more severe depressive symptoms than patients with baseline anxiety and OCD. These results are in line with research showing that individuals with more severe depression improve more than patients with mild depression after receiving psychotherapy [18,19,59]. By contrast, no differences in depressive symptoms at follow-up were found in patients with anxiety disorders and OCD between the 2 conditions, suggesting that transdiagnostic iCBT might be specially indicated for reducing depressive symptoms in patients who enter transdiagnostic iCBT with a principal diagnosis of depression. No significant predictors were found for behavioral inhibition and behavioral activation.

#### Changes in Health-Related QoL

A baseline principal diagnosis of anxiety predicted better health-related QoL at follow-up after transdiagnostic iCBT but not after TAU, which suggests that transdiagnostic iCBT might be particularly beneficial for health-related QoL in patients with primary anxiety disorders. In addition, being male and being on medication at baseline predicted improvements in health-related QoL at follow-up after transdiagnostic iCBT but not after TAU. With regard to being male, sex has not usually been reported as a predictor of outcomes in the cognitive behavioral therapy literature [60,61].

#### Changes in Diagnostic Status and Comorbidity

Age at baseline predicted changes in diagnostic status at follow-up. Specifically, older participants were more likely to not meet the diagnostic criteria for their principal baseline diagnosis at follow-up after transdiagnostic iCBT, whereas an opposite pattern was observed for older participants in the TAU group. Moreover, older individuals who received transdiagnostic iCBT also showed less comorbidity at follow-up than older individuals who received TAU. Taken together, these findings suggest that older individuals with emotional disorders are adequate targets for transdiagnostic iCBT in specialized care, at least in terms of improving their diagnostic status and comorbidity. Likewise, participants with nonuniversity and university education had significantly less comorbidity at follow-up. Moreover, participants with nonuniversity education in the transdiagnostic iCBT group had significantly less comorbidity at follow-up than participants with nonuniversity education in the TAU group. These results support the notion that, regardless of sociodemographic variables such as age and educational level, iCBT probably works equally well [21].

### Limitations

The results should be interpreted in the context of some limitations. First, attrition was high in both conditions at 1-year follow-up. However, it should be noted that data were missing completely at random. Moreover, dropout rates in internet interventions are high even at short-term follow-ups [62]. Second, the sample size was small, which may affect the representativeness of the findings achieved in this study. Third, baseline to 1-year follow-up disorder-specific symptoms (eg, panic disorder symptoms and social anxiety disorder symptoms) could not be analyzed because of the small sample size. Finally, although no differences were observed between the groups in anxiety symptoms, a noninferiority trial design would be needed to confirm that transdiagnostic iCBT was not inferior to TAU in improving anxiety symptoms in the long term.

### Conclusions

The findings show that transdiagnostic iCBT in public specialized care is in general effective for the treatment of emotional disorders in the long term. Together with our previously published RCT, we have provided research that shows both the short- and long-term effectiveness of transdiagnostic iCBT in public specialized mental health care. In addition, it should be highlighted that specialized mental health care in Spain is provided by psychiatrists and clinical psychologists with the highest degree of specialization in the treatment of psychological disorders in the national public health care system, which the authors believe adds value to the findings of this study. On the basis of these results, we encourage other researchers to conduct studies with a specific focus on implementation in this setting to achieve widespread integration of iCBT in the Spanish national public health care system, which has also been deeply affected by the consequences of the COVID-19 pandemic. In our analysis of predictors, the results showed trends suggesting that transdiagnostic iCBT predicts improvements in depression for patients with baseline depression. However, we did not find such patterns for baseline anxiety disorders and anxiety symptoms. As this is, to our knowledge, the first study to explore predictors of long-term outcomes in transdiagnostic iCBT, further studies should be conducted to shed light on the predictor role of sociodemographic and clinical variables for these interventions. Finally, in addition to sociodemographic and clinical variables, other variables specific to iCBT, such as the association between program use (eg, number of log-ins, time spent in each treatment module, and number of activities completed) and outcome in iCBT, should be further studied. Although research exists on the association among these variables, the findings mostly refer to posttreatment results [63-65]. Hence, we recommend that future research should analyze the association between program use and outcomes not only in the treatment period but also in the follow-up periods.

## Appendix

Multimedia Appendix 1 Fixed-effect parameter estimates and their corresponding 95% CIs for each predictor of long-term changes separately.

Multimedia Appendix 2 CONSORT-eHEALTH checklist (V 1.6.2).

## Abbreviations

BAI: Beck Anxiety Inventory
BAS: behavioral activation system
BDI-II: Beck Depression Inventory, Second Edition
BIS: behavioral inhibition system
DV: dependent variable
iCBT: internet-delivered cognitive behavioral therapy
OCD: obsessive-compulsive disorder
QoL: quality of life
RCT: randomized controlled trial
TAU: treatment as usual
