# Supplementary material for: Long-term Effectiveness and Predictors of Transdiagnostic Internet-Delivered Cognitive Behavioral Therapy for Emotional Disorders in Specialized Care: Secondary Analysis of a Randomized Controlled Trial
Source: JMIR Ment Health. 2022 Oct 31;9(10):e40268. doi: 10.2196/40268 (PMC9664329; doi:10.2196/40268)

**Multimedia Appendix 1.** Fixed-effect parameter estimates and their corresponding 95% confidence intervals for each predictor of long-term changes separately. Estimates with CI containing 0 indicate a not significant Group1*Time4*Predictor interaction.


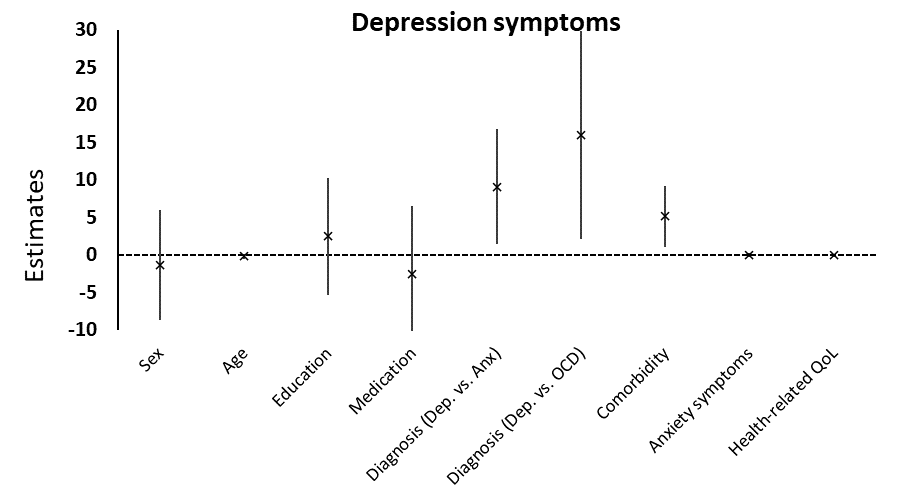


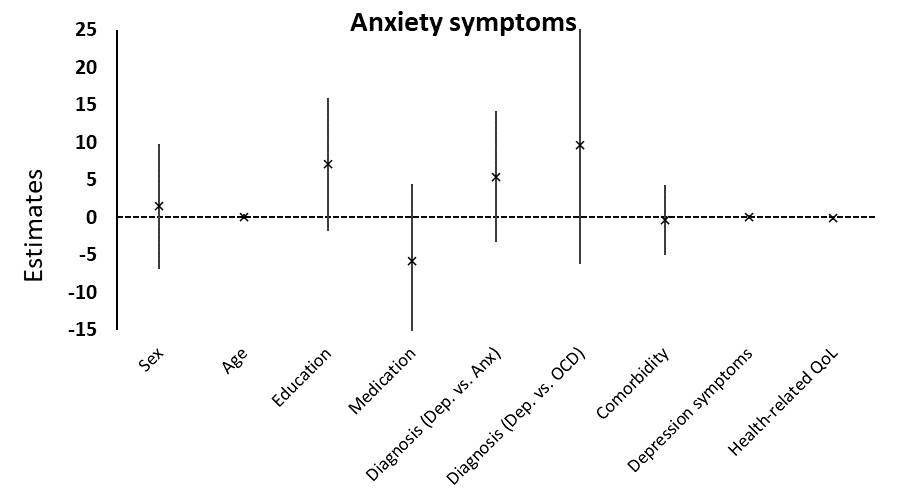


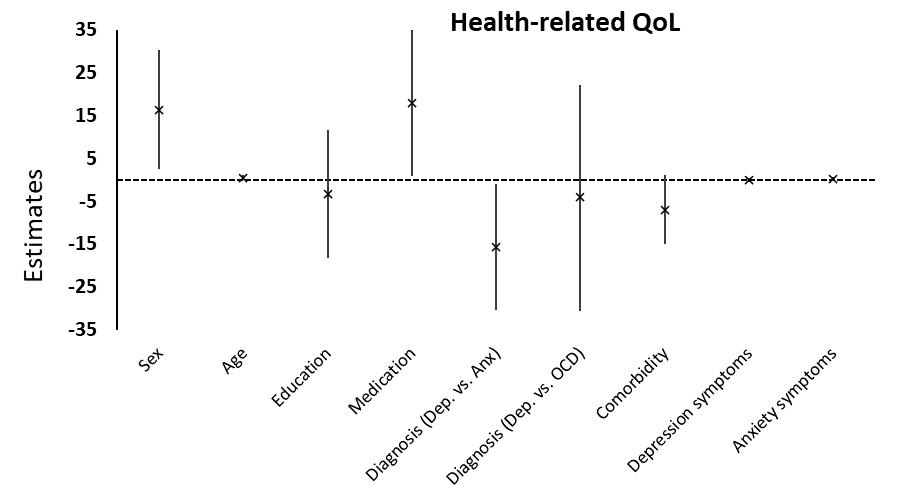


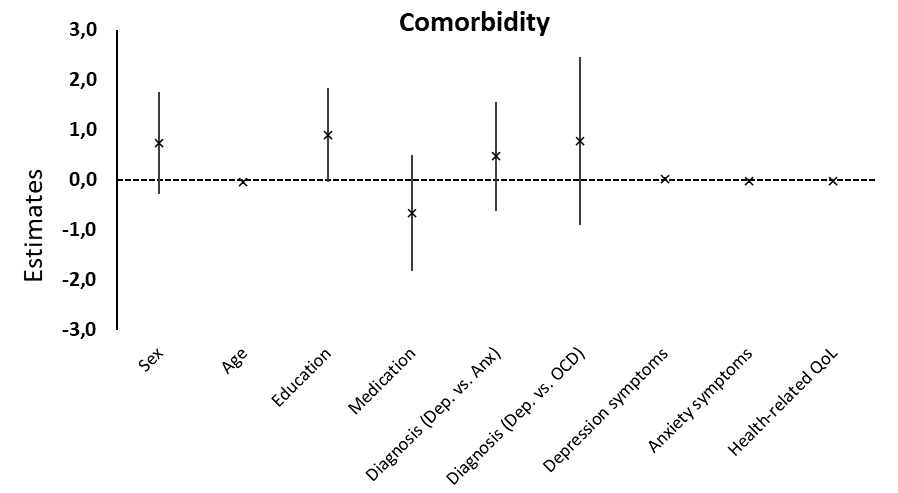


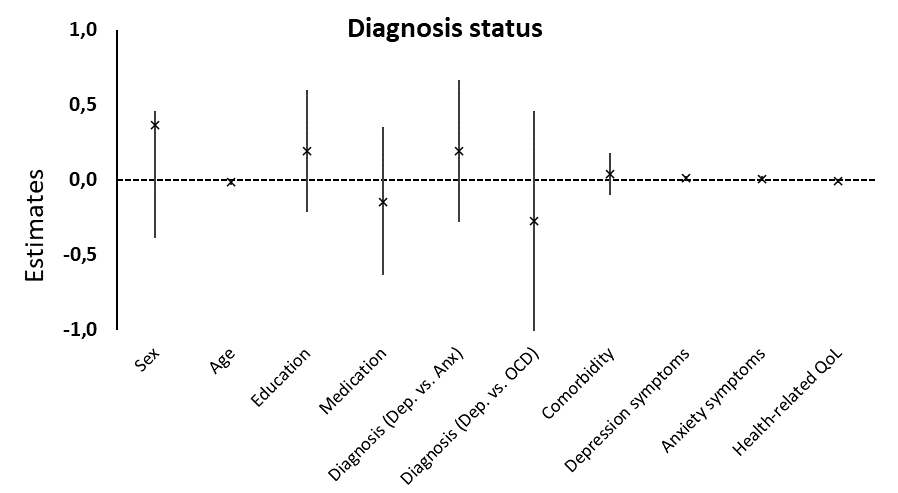


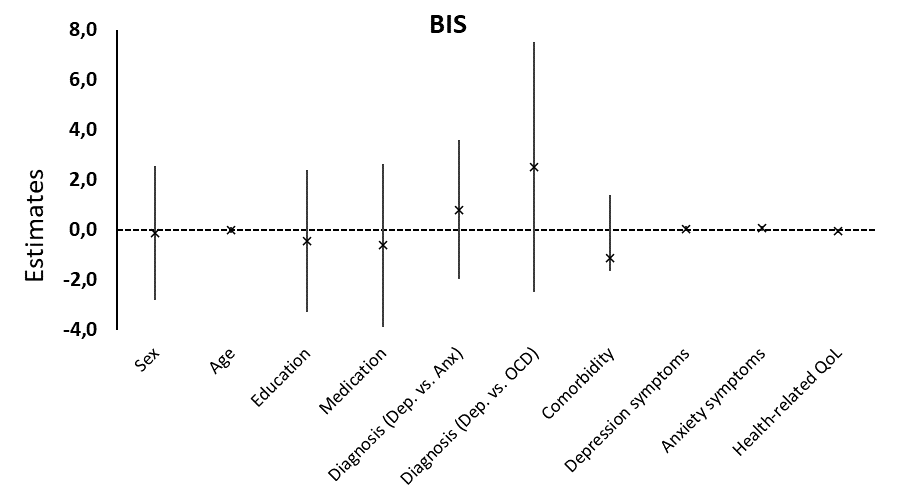


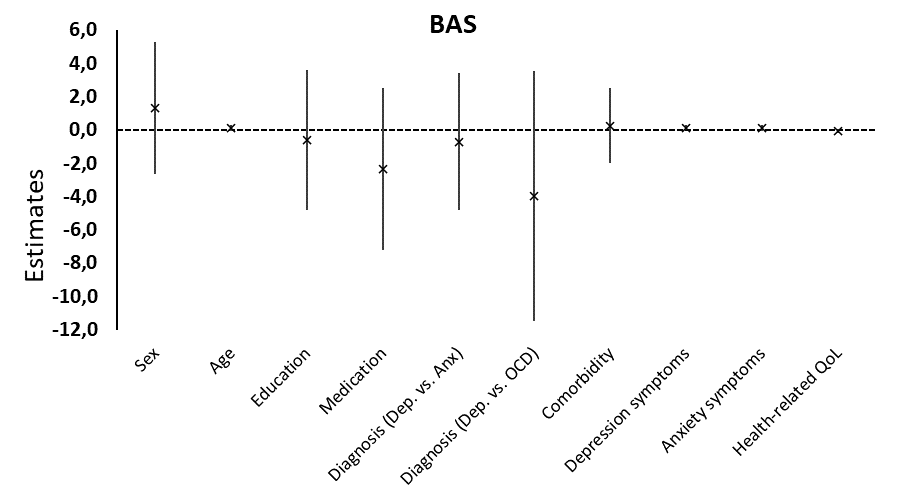

Supplement: Multimedia Appendix 1 [file mental_v9i10e40268_app1.docx]
