# Supplementary material for: Long-term Effectiveness and Predictors of Transdiagnostic Internet-Delivered Cognitive Behavioral Therapy for Emotional Disorders in Specialized Care: Secondary Analysis of a Randomized Controlled Trial
Source: JMIR Ment Health. 2022 Oct 31;9(10):e40268. doi: 10.2196/40268 (PMC9664329; doi:10.2196/40268)
Supplement: Multimedia Appendix 2 [file mental_v9i10e40268_app2.pdf]

CONSORT-EHEALTH Checklist V1.6.2 Report	Manuscript Number	40268
**(based on CONSORT-EHEALTH V1.6), available at [http://tinyurl.com/consort-ehealth-v1-6].**		
**Date completed**		
10/7/2022 5:40:20		
**by**		
Alberto		
Long-term effectiveness and predictors of transdiagnostic iCBT for emotional disorders in public specialized mental health care		
TITLE		
**1a-i) Identify the mode of delivery in the title**		
"In this study we aimed to a) analyze the long-term effectiveness of transdiagnostic iCBT compared to treatment as usual in specialized care, and b) to explore predictors of long-term effectiveness"		
**1a-ii) Non-web-based components or important co-interventions in title**		
This intervention was guided but guidance is not mentioned in this specific study. Instead, the reader is referred to the principal outcome study where guidance is fully described.		
**1a-iii) Primary condition or target group in the title**		
"Long-term effectiveness and predictors of transdiagnostic iCBT for emotional disorders in specialized care: secondary analysis of a randomized controlled trial"		
ABSTRACT		
**1b-i) Key features/functionalities/components of the intervention and comparator in the METHODS section of the ABSTRACT**		
"Objective: In this study we aimed to a) analyze the long-term effectiveness of transdiagnostic iCBT compared to treatment as usual in specialized care, and b) to explore predictors of long-term effectiveness.<br>Methods: Mixed models were performed to analyze the long-term effectiveness and predictors of transdiagnostic iCBT (EmotionRegulation) (n= 99) versus treatment as usual (n = 101) in public specialized mental health care. Outcomes included symptoms of depression and anxiety, health-related quality of life (QoL), behavioral inhibition/behavioral activation, comorbidity, and diagnostic status (i.e., loss of principal diagnosis) from baseline to one-year follow-up. Sociodemographic characteristics (sex, age, education), and clinical variables (principal diagnosis, comorbidity, and symptom severity at baseline) were selected as predictors of long-term changes."		
**1b-ii) Level of human involvement in the METHODS section of the ABSTRACT**		
This intervention was guided but guidance is not mentioned in this specific study. Instead, the reader is referred to the principal outcome study where guidance is fully described.		
**1b-iii) Open vs. closed, web-based (self-assessment) vs. face-to-face assessments in the METHODS section of the ABSTRACT**		
We refer to the reader to the study protocol paper and the main outcomes paper for a full description of these aspects.<br>"The study design of the RCT has been fully described elsewhere [27]."<br>"Additional details about this treatment have been reported [26, 27]."		
**1b-iv) RESULTS section in abstract must contain use data**		
"The flowchart of participants from baseline to one-year follow-up can be seen in Figure 1. A total of 326 patients expressed interest in the study, 281 of whom were assessed for eligibility. Of these 281 participants, 67 were excluded from the study. A total of 214 participants were randomized to either EmotionRegulation (n=106) or TAU (n=108). In addition, seven patients in each condition withdrew from the study before the pretreatment assessment, and so they were excluded from the analyses. Therefore, the final sample at baseline comprised 99 participants in EmotionRegulation and 101 participants in TAU. One-year follow-up data were obtained from 46 participants (46%) in the EmotionRegulation condition and from 47 participants (47%) in the TAU condition"		
**1b-v) CONCLUSIONS/DISCUSSION in abstract for negative trials**		
No negative outcomes were found in this study		
INTRODUCTION		
**2a-i) Problem and the type of system/solution**		
"Mixed models were performed to analyze the long-term effectiveness and predictors of transdiagnostic iCBT (EmotionRegulation) (n= 99) versus treatment as usual (n = 101) in public specialized mental health care"<br>"To the best of our knowledge, studies on the long-term effectiveness of iCBT in public specialized mental health care are scarce in the literature. However, the high demand for mental health resources along with the lack of resources in this specific setting [24, 25] highlight the need for evidence-based interventions that are also effective in the long term. In a previously published RCT, we examined the effectiveness of a transdiagnostic iCBT compared to treatment as usual (TAU) in public specialized mental health care (González-Robles et al., 2020). Transdiagnostic iCBT was found to be more effective than TAU on measures of anxiety (d= 0.35), depression (d= 0.41), and health-related QoL (d= -0.45) at post-treatment. However, the effects of this intervention in the long term (1-year follow-up) have not yet been analyzed. Therefore, the aim of the present investigation was twofold: a) to analyze the long-term outcomes of transdiagnostic iCBT for emotional disorders, compared to TAU; and b) to analyze potential predictors of the long-term effectiveness of transdiagnostic iCBT versus TAU."		
**2a-ii) Scientific background, rationale: What is known about the (type of) system**		
"Among the range of iCBT programs, transdiagnostic iCBT for emotional disorders has shown its efficacy and effectiveness in a growing number of randomized controlled trials (RCT). Several meta-analyses have shown that transdiagnostic iCBT is effective in the short term, with pooled effect sizes (Hedges g) in the medium to large range for overall measures of anxiety (0.78-0.82), depression (0.79-0.84), and quality of life (QoL) (0.48-0.56) at post-treatment [13, 14]. However, most of these available meta-analytic studies mainly report post-treatment outcomes, which suggests that more research is needed on long-term effects of transdiagnostic iCBT."		
**Does your paper address CONSORT subitem 2b?**		
"Therefore, the aim of the present investigation was twofold: a) to analyze the long-term outcomes of transdiagnostic iCBT for emotional disorders, compared to TAU; and b) to analyze potential predictors of the long-term effectiveness of transdiagnostic iCBT versus TAU"		
METHODS		
**3a) CONSORT: Description of trial design (such as parallel, factorial) including allocation ratio**		
"The current study analyzes long-term data (1-year follow-up) on a previously published RCT that compared transdiagnostic iCBT to treatment as usual in public specialized mental healthcare services [26]."		
**3b) CONSORT: Important changes to methods after trial commencement (such as eligibility criteria), with reasons**		
No changes in the methods have happened after trial commencement.		
**3b-i) Bug fixes, Downtimes, Content Changes**		
There are no unexpected events to report in this study.		
**4a) CONSORT: Eligibility criteria for participants**		
"To participate in the study, patients had to meet the following eligibility criteria: (1) aged 18 years or older; (2) ability to understand and read Spanish; (3) access to the Internet at home and an email address; (4) fulfill Diagnostic and Statistical Manual of Mental Disorders, Fourth Edition (DSM-IV-TR) diagnostic criteria [28] for ED, including major depressive disorder, dysthymic disorder, depression not otherwise specified, panic disorder, agoraphobia, social anxiety disorder, generalized anxiety disorder, anxiety not otherwise specified, and obsessive-compulsive disorder; (5) provide written informed consent; (6) absence of schizophrenia, bipolar disorder, and alcohol and/or substance dependence disorder; (7) absence of high risk of suicide; (8) absence of a disabling medical disease that prevented the participant from carrying out the psychological treatment; and (9) not receiving another psychological treatment during the study (in the experimental group). Pharmacological treatment was allowed, but participants had to be taking the same dose during the two months prior to enrolling in the study. In addition, participants in the experimental group whose medication was increased or changed during the study period were excluded from the trial (decreases in pharmacological treatment were accepted)."		
**4a-i) Computer / Internet literacy**		
This aspect is not included in the eligibility criteria.		
**4a-ii) Open vs. closed, web-based vs. face-to-face assessments:**		
"Participants were adults who attended public mental health units in Spain to seek psychological and/or psychiatric help between July 2015 and June 2019. Potential participants were identified by the psychiatrists and psychologists in these centers and referred to the study researchers for eligibility assessment (see González-Robles et al., 2020 for a full description of the recruitment process)."		
**4a-iii) Information giving during recruitment**		
Participants were briefed for recruitment. However, this information is not provided either in any of the previous published studies related to this paper.		
**4b) CONSORT: Settings and locations where the data were collected**		
"Participants were adults who attended public mental health units in Spain to seek psychological and/or psychiatric help between July 2015 and June 2019. Potential participants were identified by the psychiatrists and psychologists in these centers and referred to the study researchers for eligibility assessment (see González-Robles et al., 2020 for a full description of the recruitment process)."		
**4b-i) Report if outcomes were (self-)assessed through online questionnaires**		
This aspect is not mentioned in the current paper but the reader is referred to the study protocol and main outcomes paper for a description of these aspects.<br>"The study design of the RCT has been fully described elsewhere [27]."<br>"(see González-Robles et al., 2020 for a full description of the recruitment process)"		
**4b-ii) Report how institutional affiliations are displayed**		

**5) CONSORT: Describe the interventions for each group with sufficient details to allow replication, including how and when they were actually administered**		
**5-i) Mention names, credential, affiliations of the developers, sponsors, and owners**		
**5-ii) Describe the history/development process**		
**5-iii) Revisions and updating**		
**5-iv) Quality assurance methods**		
**5-v) Ensure replicability by publishing the source code, and/or providing screenshots/screen-capture video, and/or providing flowcharts of the algorithms used**		
**5-vi) Digital preservation**		
**5-vii) Access**		
This aspect is not mentioned in the current paper but the reader is referred to the study protocol and main outcomes paper for a description of these aspects.<br>"The study design of the RCT has been fully described elsewhere [27]."<br>"(see González-Robles et al., 2020 for a full description of the recruitment process)"		
**5-viii) Mode of delivery, features/functionalities/components of the intervention and comparator, and the theoretical framework**		
"All participants received a 12-module transdiagnostic iCBT protocol (EmotionRegulation) through the web platform a web platform design by our research group [36]. The core components of the treatment are based on the Unified Protocol [37, 38] and some treatment strategies from Dialectical Behavioral Therapy (e.g., what and how techniques) [39]. Participants are trained to learn and practice adaptive emotion regulation skills through the following components: a) present-focused emotional awareness (Modules 4 and 5); b) cognitive flexibility (Modules 6 and 7); c) identification and modification of emotional avoidance patterns and emotion-driven behaviors (Modules 8 and 9); and d) exposure (interoceptive and situational) (Modules 10 and 11). The treatment contains four additional modules: an introduction module (Module 1), a module to facilitate the patient's engagement with the therapy (Module 2), a module with psychoeducation on emotions (Module 3), and a relapse prevention module at the end of the treatment (Module 12). In addition, EmotionRegulation includes a Module 0 (Welcome module) with information and recommendations about how to use the protocol. The modules are presented sequentially to enable step-by-step movement through the program. All participants had access to the protocol for a maximum period of 18 weeks, and they were allowed to use the program any time they wanted to during the trial period (i.e., including the follow-up periods). Additional details about this treatment have been reported [26, 27]. The treatment modules and their goals are depicted in Table 1."		
**5-ix) Describe use parameters**		
This aspect is not mentioned in the current paper but the reader is referred to the study protocol and main outcomes paper for a description of these aspects.<br>"The study design of the RCT has been fully described elsewhere [27]."<br>"(see González-Robles et al., 2020 for a full description of the recruitment process)"		
**5-x) Clarify the level of human involvement**		
This aspect is not mentioned in the current paper but the reader is referred to the study protocol and main outcomes paper for a description of these aspects.<br>"The study design of the RCT has been fully described elsewhere [27]."<br>"(see González-Robles et al., 2020 for a full description of the recruitment process)"		
**5-xi) Report any prompts/reminders used**		
This aspect is not mentioned in the current paper but the reader is referred to the study protocol and main outcomes paper for a description of these aspects.<br>"The study design of the RCT has been fully described elsewhere [27]."<br>"(see González-Robles et al., 2020 for a full description of the recruitment process)"		
**5-xii) Describe any co-interventions (incl. training/support)**		
This aspect is not mentioned in the current paper but the reader is referred to the study protocol and main outcomes paper for a description of these aspects.<br>"The study design of the RCT has been fully described elsewhere [27]."<br>"(see González-Robles et al., 2020 for a full description of the recruitment process)"		
**6a) CONSORT: Completely defined pre-specified primary and secondary outcome measures, including how and when they were assessed**		
"Principal outcomes<br>Beck Depression Inventory, Second Edition (BDI-II) [29]. The BDI-II is a self-report scale consisting of 21 items about the symptoms that characterize MDD. Scores on each item range from 0 to 3, and the maximum score is 63 points. The instrument has demonstrated internal consistency in both the original version (α=.76-.95) and the Spanish version (α=.87 to .89) [30]. Cronbach α for the BDI-II in this study at baseline was .90.<br>Beck Anxiety Inventory (BAI) [31] is a 21-item self-report questionnaire that assesses anxiety, with a maximum score of 63 points. Each item has a 4-point severity scale (from 0: not at all to 3: severely) that addresses anxiety symptoms experienced during the previous week. Several validation studies have shown adequate psychometric properties, with good to excellent internal consistency (α between 0.85 and 0.94) and convergent and divergent validity. The Spanish version of the BAI has demonstrated high internal consistency (α=.93) [32]. Cronbach α for the BAI in this study at baseline was .92.<br><br>Secondary outcomes<br>Quality of Life EuroQoL-5D-3L Questionnaire (EQ-5D-3L) [33] is a generic instrument that measures health-related quality of life (QoL) and consists of two parts. Part 1 assesses self-reported problems in each of the following five domains: mobility, self-care, daily activities, pain/discomfort, and anxiety/depression. Part 2 records the subject's self-assessed health on a visual analog scale (VAS), a 10-cm vertical line on which the best and worst imaginable health states score 100 and 0, respectively. In this study, health-related QoL was assessed using the VAS.<br>Behavioral Inhibition Scale and Behavioral Activation Scale (BIS/BAS) [34, 35] contains 20 items rated from 1 to 4, with seven BIS subscale items that evaluate individuals' emotional responses to impending negative events and 13 BAS items that evaluate individuals' behavioral and emotional responses to potentially positive events. The BIS and BAS have shown good reliability in individuals with ED (α= .73-.92) and good convergent and discriminant validity as indicators of temperament. The internal consistency of the Spanish version ranges between 0.65 and 0.82. Cronbach α for the BIS and BAS subscales in this study at baseline were .61 and .80, respectively."		
**6a-i) Online questionnaires: describe if they were validated for online use and apply CHERRIES items to describe how the questionnaires were designed/deployed**		
**6a-ii) Describe whether and how "use" (including intensity of use/dosage) was defined/measured/monitored**		
This aspect is not reported in the current paper. See González-Robles et al. (2021) for a full description of how use was measured and analyzed.		
**6a-iii) Describe whether, how, and when qualitative feedback from participants was obtained**		
**6b) CONSORT: Any changes to trial outcomes after the trial commenced, with reasons**		
"Participants were adults who attended public mental health units in Spain to seek psychological and/or psychiatric help between July 2015 and June 2019. Potential participants were identified by the psychiatrists and psychologists in these centers and referred to the study researchers for eligibility assessment (see González-Robles et al., 2020 for a full description of the recruitment process)."		
**7a) CONSORT: How sample size was determined**		
**7a-i) Describe whether and how expected attrition was taken into account when calculating the sample size**		
This aspect is not mentioned in the current paper but the reader is referred to the study protocol and main outcomes paper for a description of these aspects.<br>"The study design of the RCT has been fully described elsewhere [27]."<br>"(see González-Robles et al., 2020 for a full description of the recruitment process)"		
**7b) CONSORT: When applicable, explanation of any interim analyses and stopping guidelines**		

"Principal outcomes<br>Beck Depression Inventory, Second Edition (BDI-II) [29]. The BDI-II is a self-report scale consisting of 21 items about the symptoms that characterize MDD. Scores on each item range from 0 to 3, and the maximum score is 63 points. The instrument has demonstrated internal consistency in both the original version (α=.76-.95) and the Spanish version (α=.87 to .89) [30]. Cronbach α for the BDI-II in this study at baseline was .90.<br>Beck Anxiety Inventory (BAI) [31] is a 21-item self-report questionnaire that assesses anxiety, with a maximum score of 63 points. Each item has a 4-point severity scale (from 0: not at all to 3: severely) that addresses anxiety symptoms experienced during the previous week. Several validation studies have shown adequate psychometric properties, with good to excellent internal consistency (α between 0.85 and 0.94) and convergent and divergent validity. The Spanish version of the BAI has demonstrated high internal consistency (α=.93) [32]. Cronbach α for the BAI in this study at baseline was .92.<br><br>Secondary outcomes<br>Quality of Life EuroQoL-5D-3L Questionnaire (EQ-5D-3L) [33] is a generic instrument that measures health-related quality of life (QoL) and consists of two parts. Part 1 assesses self-reported problems in each of the following five domains: mobility, self-care, daily activities, pain/discomfort, and anxiety/depression. Part 2 records the subject's self-assessed health on a visual analog scale (VAS), a 10-cm vertical line on which the best and worst imaginable health states score 100 and 0, respectively. In this study, health-related QoL was assessed using the VAS.<br>Behavioral Inhibition Scale and Behavioral Activation Scale (BIS/BAS) [34, 35] contains 20 items rated from 1 to 4, with seven BIS subscale items that evaluate individuals' emotional responses to impending negative events and 13 BAS items that evaluate individuals' behavioral and emotional responses to potentially positive events. The BIS and BAS have shown good reliability in individuals with ED (α= .73-.92) and good convergent and discriminant validity as indicators of temperament. The internal consistency of the Spanish version ranges between 0.65 and 0.82. Cronbach α for the BIS and BAS subscales in this study at baseline were .61 and .80, respectively."		
**8a) CONSORT: Method used to generate the random allocation sequence**		
This aspect is not mentioned in the current paper but the reader is referred to the study protocol and main outcomes paper for a description of these aspects.<br>"The study design of the RCT has been fully described elsewhere [27]."<br>"(see González-Robles et al., 2020 for a full description of the recruitment process)"		
**8b) CONSORT: Type of randomisation; details of any restriction (such as blocking and block size)**		
This aspect is not mentioned in the current paper but the reader is referred to the study protocol and main outcomes paper for a description of these aspects.<br>"The study design of the RCT has been fully described elsewhere [27]."<br>"(see González-Robles et al., 2020 for a full description of the recruitment process)"		
**9) CONSORT: Mechanism used to implement the random allocation sequence (such as sequentially numbered containers), describing any steps taken to conceal the sequence until interventions were assigned**		
This aspect is not mentioned in the current paper because it is a secondary analysis but the reader is referred to the study protocol and main outcomes paper for a description of these aspects.<br>"The study design of the RCT has been fully described elsewhere [27]."<br>"(see González-Robles et al., 2020 for a full description of the recruitment process)"		
**10) CONSORT: Who generated the random allocation sequence, who enrolled participants, and who assigned participants to interventions**		
This aspect is not mentioned in the current paper because it is a secondary analysis but the reader is referred to the study protocol and main outcomes paper for a description of these aspects.<br>"The study design of the RCT has been fully described elsewhere [27]."<br>"(see González-Robles et al., 2020 for a full description of the recruitment process)"		
**11a) CONSORT: Blinding - If done, who was blinded after assignment to interventions (for example, participants, care providers, those assessing outcomes) and how**		
**11a-i) Specify who was blinded, and who wasn't**		
This aspect is not mentioned in the current paper but the reader is referred to the study protocol and main outcomes paper for a description of these aspects.<br>"The study design of the RCT has been fully described elsewhere [27]."<br>"(see González-Robles et al., 2020 for a full description of the recruitment process)"		
**11a-ii) Discuss e.g., whether participants knew which intervention was the "intervention of interest" and which one was the "comparator"**		
This aspect is not mentioned in the current paper but the reader is referred to the study protocol and main outcomes paper for a description of these aspects.<br>"The study design of the RCT has been fully described elsewhere [27]."<br>"(see González-Robles et al., 2020 for a full description of the recruitment process)"		
**11b) CONSORT: If relevant, description of the similarity of interventions**		
Not applicable because the comparator was treatment as usual.		
**12a) CONSORT: Statistical methods used to compare groups for primary and secondary outcomes**		
"Mixed-effects models were conducted to analyze the long-term effects and predictors of EmotionRegulation using the lmer function from the lme4 R-package [41], with R version 4.0.2 [42]. Analyses were conducted via Restricted Maximum Likelihood estimation (REML) [43, 44]. In contrast to multiple imputation methods (i.e., to fill in missing data) or complete-case data through detection methods (which results in biased estimations), the REML method allows incomplete/unbalanced data to be modeled by finding parameters that maximize the likelihood using all the available data, providing a less-biased estimate of variance components with smaller sample sizes [43, 45, 46]. To compute the magnitude of between-group changes at one-year follow-up, effect sizes (Cohen's d) were calculated by dividing the differences in means by the pooled SD. Effect sizes were interpreted according to Cohen's convention: effect sizes of 0.20 are considered low, effect sizes of 0.50 are considered medium, and effect sizes of 0.80 and above are considered large [47]."		
**12a-i) Imputation techniques to deal with attrition / missing values**		
"In contrast to multiple imputation methods (i.e., to fill in missing data) or complete-case data through detection methods (which results in biased estimations), the REML method allows incomplete/unbalanced data to be modeled by finding parameters that maximize the likelihood using all the available data, providing a less-biased estimate of variance components with smaller sample sizes [43, 45, 46]. "		
**12b) CONSORT: Methods for additional analyses, such as subgroup analyses and adjusted analyses**		
We analyzed predictors of long-term effectivenes: "Mixed-effects models were conducted to analyze the long-term effects and predictors of EmotionRegulation using the lmer function from the lme4 R-package [41], with R version 4.0.2 [42]." "Furthermore, we selected different baseline variables as potential predictors of long-term outcomes, including demographic variables (i.e., sex [0=men; 1=women], age, and education [0=non-university (basic and medium studies); 1=university]), clinical status (i.e., medication [0=no; 1=yes], principal diagnosis [0=depression; 1=anxiety; 2=OCD], comorbidity (number of clinical diagnoses), and diagnostic status [0=does not meet diagnostic criteria; 1= meets diagnostic criteria]), dispositional traits (i.e., behavioral inhibition [BI] and behavioral activation [BA]), and symptomatology (anxiety [BAI], depression [BDI-II], and health-related QoL [EQ-5D-5L]). Given that we were interested in long-term changes (i.e., one-year follow-up relative to baseline), we focused on Group1*Time4 interactions (i.e., EmotionRegulation*1-year follow-up) to analyze the long-term effects and predictors [48]."		
RESULTS		
**13a) CONSORT: For each group, the numbers of participants who were randomly assigned, received intended treatment, and were analysed for the primary outcome**		
"The flowchart of participants from baseline to one-year follow-up can be seen in Figure 1. A total of 326 patients expressed interest in the study, 281 of whom were assessed for eligibility. Of these 281 participants, 67 were excluded from the study. A total of 214 participants were randomized to either EmotionRegulation (n=106) or TAU (n=108). In addition, seven patients in each condition withdrew from the study before the pretreatment assessment, and so they were excluded from the analyses. Therefore, the final sample at baseline comprised 99 participants in EmotionRegulation and 101 participants in TAU. One-year follow-up data were obtained from 46 participants (46%) in the EmotionRegulation condition and from 47 participants (47%) in the TAU condition. "		
**13b) CONSORT: For each group, losses and exclusions after randomisation, together with reasons**		
This aspect is not mentioned in the current paper but the reader is referred to the main outcomes paper for a description of these aspects.<br>"The study design of the RCT has been fully described elsewhere [27]."<br>"(see González-Robles et al., 2020 for a full description of the recruitment process)"		
**13b-i) Attrition diagram**		
**14a) CONSORT: Dates defining the periods of recruitment and follow-up**		
This aspect is not mentioned in the current paper but the reader is referred to the study protocol and main outcomes paper for a description of these aspects.<br>"The study design of the RCT has been fully described elsewhere [27]."<br>"(see González-Robles et al., 2020 for a full description of the recruitment process)"		
**14a-i) Indicate if critical "secular events" fell into the study period**		
**14b) CONSORT: Why the trial ended or was stopped (early)**		
This item is not applicable because the trial did not end or stopped early.		
**15) CONSORT: A table showing baseline demographic and clinical characteristics for each group**		
The manuscript includes a table that contains demographic and clinical characteristics of the sample in the results section (Baseline data) (Table 2).		
**15-i) Report demographics associated with digital divide issues**		
The manuscript includes a table that contains these types of demographic data in the results section (Baseline data) (Table 2).		

**16a) CONSORT: For each group, number of participants (denominator) included in each analysis and whether the analysis was by original assigned groups**		
**16-i) Report multiple “denominators” and provide definitions**		
Not applicable to this study.		
**16-ii) Primary analysis should be intent-to-treat**		
"In contrast to multiple imputation methods (i.e., to fill in missing data) or complete-case data through detection methods (which results in biased estimations), the REML method allows incomplete/unbalanced data to be modeled by finding parameters that maximize the likelihood using all the available data, providing a less-biased estimate of variance components with smaller sample sizes [43, 45, 46]. "		
**17a) CONSORT: For each primary and secondary outcome, results for each group, and the estimated effect size and its precision (such as 95% confidence interval)**		
Sample sizes are provided for primary and secondary outcomes in each group, including 95% confidence intervals (Table 3).		
**17a-i) Presentation of process outcomes such as metrics of use and intensity of use**		
This aspect is not reported in the current paper. See González-Robles et al. (2021) for a full description of how use was measured and analyzed.		
**17b) CONSORT: For binary outcomes, presentation of both absolute and relative effect sizes is recommended**		
Not applicable to this study.		
**18) CONSORT: Results of any other analyses performed, including subgroup analyses and adjusted analyses, distinguishing pre-specified from exploratory**		
"Fixed-effect parameter estimates and their corresponding 95% confidence intervals for each predictor of long-term changes separately are shown in Supplementary file 1. As indicated previously, we first conducted univariate mixed models to investigate the independent contribution of each potential predictor of long-term changes separately. Significant predictors in univariate models were then entered simultaneously into a multivariate mixed model. In the following paragraphs we report the results on predictors for the following variables are reported: a) depressive and anxiety symptoms, and behavioral inhibition/activation; b) health-related QoL; c) diagnosis status; and d) comorbidity."		
**18-i) Subgroup analysis of comparing only users**		
**19) CONSORT: All important harms or unintended effects in each group**		
Deterioration rates were published in the main outcomes paper and are not relevant to the current paper.		
**19-i) Include privacy breaches, technical problems**		
**19-ii) Include qualitative feedback from participants or observations from staff/researchers**		
DISCUSSION		
**20) CONSORT: Trial limitations, addressing sources of potential bias, imprecision, multiplicity of analyses**		
**20-i) Typical limitations in ehealth trials**		
"The results should be interpreted in the context of some limitations. First, attrition was high in both conditions at one-year follow-up. It should be noted, though, that missing data were completely at random. Moreover, dropout rates in Internet interventions are high even at short-term follow-ups [62]. Second, the sample size was small, which may affect the representativeness of the findings achieved in this study. Third, baseline to 1-year follow-up disorder-specific symptoms (e.g., panic disorder symptoms, social anxiety disorder symptoms) could not be analyzed due to small sample size. Finally, although no differences were observed between groups in anxiety symptoms, a non-inferiority trial design would be needed to confirm that transdiagnostic iCBT was not inferior to TAU in improving anxiety symptoms in the long-term. "		
**21) CONSORT: Generalisability (external validity, applicability) of the trial findings**		
**21-i) Generalizability to other populations**		
**21-ii) Discuss if there were elements in the RCT that would be different in a routine application setting**		
**22) CONSORT: Interpretation consistent with results, balancing benefits and harms, and considering other relevant evidence**		
**22-i) Restate study questions and summarize the answers suggested by the data, starting with primary outcomes and process outcomes (use)**		
"Taken together, EmotionRegulation was shown to be more effective than TAU at one-year follow-up for the treatment of emotional disorders in specialized care. Specifically, in comparison with TAU, EmotionRegulation was more effective in reducing symptoms of depression and improving health-related QoL at one-year follow-up, with effect sizes in the small/moderate range. Furthermore, participants in EmotionRegulation had a better diagnostic status (i.e., 22% of patients in EmotionRegulation met the diagnosis at one-year follow-up, versus 46% of patients in TAU) and showed less comorbidity (0.7 diagnoses in EmotionRegulation versus 1.38 diagnoses in TAU). Regarding anxiety, both groups improved their scores from pre-treatment to one-year follow-up, without significant differences between groups. These results suggests that EmotionRegulation was, at least, as effective as treatment as usual for anxiety symptoms in the long term."		
**22-ii) Highlight unanswered new questions, suggest future research**		
"Another noteworthy finding is that the scores on behavioral inhibition were significantly lower in patients from the iCBT transdiagnostic condition, with an effect size in the large range (d= 0.87), while no differences were found for behavioral activation. The study of how these and other related dimensions (e. g., neuroticism and extraversion; positive and negative affect) change following treatment is of paramount importance in the context of transdiagnostic treatments that target shared psychopathological processes [55, 56]. Nevertheless, these results can be compared to those obtained in trials that measure close constructs, such as positive and negative affect [57]. The pattern of results that we obtained in behavioral inhibition and behavioral activation resembles the pattern of results in negative and positive effect after transdiagnostic iCBT found in the literature, i.e., large gains in negative affect and low to moderate gains in positive affect [55]. With the exclusion of a study [58], to our knowledge no other studies on transdiagnostic iCBT have included this measure in their trials. Future research may benefit of including measures of behavioral inhibition/activation to study the potential of transdiagnostic iCBT in successfully addressing these specific temperament dimensions."<br>"Based on these results, we encourage other researchers to conduct studies with a specific focus on implementation in this setting to achieve widespread integration of iCBT in the Spanish Public National Health System, which has also been deeply impacted by the consequences of the covid-19 pandemic"<br>"Finally, in addition to sociodemographic and clinical variables, other variables specific to iCBT, such as the association between program usage (e. g., number of logins, time spent in each treatment module, number of activities completed and so on) and outcome in iCBT should be further studied. Although research exists on the association between these variables, their findings mostly refer to post-treatment results [63-65]. Hence, we recommend that future research should analyze the association between program usage and outcomes not only in the treatment period, but also in the follow-up periods."		
Other information		
**23) CONSORT: Registration number and name of trial registry**		
"Trial Registration: ClinicalTrials.gov NCT02345668, 27 July 2015"		
**24) CONSORT: Where the full trial protocol can be accessed, if available**		
"The study design of the RCT has been fully described elsewhere [27]."		
**25) CONSORT: Sources of funding and other support (such as supply of drugs), role of funders**		
"The authors would like to thank Consorcio Hospitalario Provincial de Castellón, Hospital Universitario de la Ribera, and Hospital Universitario Vall d'Hebrón for their invaluable contribution to this study. This research was conducted with the financial support of grants P1-1B2014-43 (Universitat Jaume I, Castellón), a PhD grant from the Ministry of Education, Culture and Sports (FPU13/00576), and CIBER Fisiopatología de la Obesidad y Nutrición-ISCIII CB06/03/0052. The funders had no role in the study design, data collection and analysis, decision to publish, or preparation of the manuscript. "		
**X26-i) Comment on ethics committee approval**		
"The protocol of the original study was registered at ClinicalTrials.gov (NCT02345668) and obtained ethical approval from the Ethics Committee of Universitat Jaume I (Castellón, Spain) and the Clinical Research Ethics Committees of the three hospitals that participated in the trial (Consorcio Hospitalario Provincial de Castellón, Hospital Universitario de la Ribera, and Hospital Universitario Vall d'Hebron)."		
**x26-ii) Outline informed consent procedures**		
This aspect is not mentioned in the current paper but the reader is referred to the study protocol and main outcomes paper for a description of these aspects.<br>"The study design of the RCT has been fully described elsewhere [27]."<br>"(see González-Robles et al., 2020 for a full description of the recruitment process)"		
**X26-iii) Safety and security procedures**		
This aspect is not mentioned in the current paper but the reader is referred to the study protocol and main outcomes paper for a description of these aspects.<br>"The study design of the RCT has been fully described elsewhere [27]."<br>"(see González-Robles et al., 2020 for a full description of the recruitment process)"		
**X27-i) State the relation of the study team towards the system being evaluated**		
This aspect is not mentioned in the current paper but the reader is referred to the study protocol and main outcomes paper for a description of these aspects.<br>"The study design of the RCT has been fully described elsewhere [27]."<br>"(see González-Robles et al., 2020 for a full description of the recruitment process)"		
